# Restless Legs Syndrome Prevalence and Clinical Correlates Among Psychiatric Inpatients: A Multicenter Study

**DOI:** 10.3389/fpsyt.2022.846165

**Published:** 2022-03-14

**Authors:** Franziska C. Weber, Heidi Danker-Hopfe, Ezgi Dogan-Sander, Lukas Frase, Anna Hansel, Nicole Mauche, Christian Mikutta, Diana Nemeth, Kneginja Richter, Claudia Schilling, Martina Sebestova, Marian M. Spath, Christoph Nissen, Thomas C. Wetter

**Affiliations:** ^1^Department of Psychiatry and Psychotherapy, University of Regensburg, Regensburg, Germany; ^2^Charité-Universitätsmedizin, Corporate Member of Freie Universität Berlin, Humboldt-Universität zu Berlin, Berlin Institute of Health, Competence Center of Sleep Medicine Berlin, Berlin, Germany; ^3^Department of Psychiatry and Psychotherapy, University of Leipzig Medical Center, Leipzig, Germany; ^4^Department of Psychiatry and Psychotherapy, Medical Center, University of Freiburg – Faculty of Medicine, University of Freiburg, Freiburg, Germany; ^5^Department of Psychiatry and Psychotherapy, University Leipzig, Medical Faculty, Leipzig, Germany; ^6^University Hospital of Psychiatry and Psychotherapy, University of Bern, Bern, Switzerland; ^7^Privatklinik Meiringen, Meiringen, Switzerland; ^8^Department of Psychiatry and Psychotherapy, Paracelsus Medical University, Nuremberg, Germany; ^9^Central Institute of Mental Health, Department of Psychiatry and Psychotherapy, Medical Faculty Mannheim, Heidelberg University, Mannheim, Germany

**Keywords:** restless legs syndrome, RLS, prevalence, psychiatric disorders, sleep quality, multicenter study, psychotropic drugs

## Abstract

**Background:**

There are only limited reports on the prevalence of restless legs syndrome (RLS) in patients with psychiatric disorders. The present study aimed to evaluate the prevalence and clinical correlates in psychiatric inpatients in Germany and Switzerland.

**Methods:**

This is a multicenter cross-sectional study of psychiatric inpatients with an age above 18 years that were diagnosed and evaluated face-to-face using the International RLS Study Group criteria (IRLSSG) and the International RLS severity scale (IRLS). In addition to sociodemographic and biometric data, sleep quality and mood were assessed using the Pittsburgh Sleep Quality Index (PSQI), the Insomnia Severity Index (ISI), the Epworth Sleepiness Scale (ESS), and the Patient Health Questionnaire (PHQ-9). In addition to univariate statistics used to describe and statistically analyze differences in variables of interest between patients with and without RLS, a logistic model was employed to identify predictors for the occurrence of RLS.

**Results:**

The prevalence of RLS in a sample of 317 psychiatric inpatients was 16.4%, and 76.9% of these were diagnosed with RLS for the first time. RLS severity was moderate to severe (IRLS ± SD: 20.3 ± 8.4). The prevalences in women (*p* = 0.0036) and in first-degree relatives with RLS (*p* = 0.0108) as well as the body mass index (BMI, *p* = 0.0161) were significantly higher among patients with RLS, while alcohol consumption was significantly lower in the RLS group. With the exception of atypical antipsychotics, treatment with psychotropic drugs was not associated with RLS symptoms. Regarding subjective sleep quality and mood, scores of the PSQI (*p* = 0.0007), ISI (*p* = 0.0003), and ESS (*p* = 0.0005) were higher in patients with RLS, while PHQ-9 scores were not different. A logistic regression analysis identified gender (OR 2.67; 95% CI [1.25; 5.72]), first-degree relatives with RLS (OR 3.29; 95% CI [1.11; 9.73], ESS score (OR 1.09; 95% CI [1.01; 1.17]), and rare alcohol consumption (OR 0.45; 95% CI [0.22; 0.94] as predictors for RLS.

**Conclusions:**

Clinically significant RLS had a high prevalence in psychiatric patients. RLS was associated with higher BMI, impaired sleep quality, and lower alcohol consumption. A systematic assessment of restless legs symptoms might contribute to improve the treatment of psychiatric patients.

## Introduction

Restless legs syndrome (RLS), also known as Willis–Ekbom disease, is a common neurological sensorimotor disorder often associated with severe sleep disturbances and an impaired quality of life (1). The reported RLS prevalence varies between 3.9 and 14.3% depending on the population studied and the criteria used (2). Some but not all reports suggest marked geographic differences in the prevalence of RLS with a lower prevalence in Asian countries (3–5). If the former four minimal (6) or later revised four essential diagnostic criteria are used (7), RLS is reported in 7.2–10.6% of the Caucasian population (8–14). When so-called RLS mimics are excluded, significantly lower prevalence rates of 0.4–2.4% are seen in different samples (15, 16). The proportion of patients in need of treatment is estimated to vary between 1.5 and 2.7% (15). In two prospective studies with observation periods of 2 and 5.2 years, cumulative incidences of 7.0 and 9.1%, respectively, were reported (17). Women are affected up to twice as often as men presumably due to parity; the risk of developing RLS increases with increasing number of births (3, 9, 18). Risk factors for RLS include genetic predisposition, age, gender, parity, and lifestyle factors (9, 19). Secondary or comorbid RLS may occur as a result of another condition such as iron deficiency, renal disease, or pregnancy (20). In addition, secondary RLS can be induced by psychotropic drugs and other medications (21–23). Current hypotheses on the etiology of RLS favor a heterogeneously genetically determined, complex disorder with involvement of abnormal iron metabolism, multiple neurotransmitters, and the opioid system. As revealed by neuroimaging and neurophysiological studies, a subtle receptor dysfunction of the central dopaminergic system seems to play a critical role in the pathophysiology of RLS (24). Clinically, levodopa and dopamine receptor agonists improve RLS, whereas dopamine antagonists such as antipsychotics may evoke or worsen RLS suggesting a decreased dopaminergic state (25). Furthermore, tyrosine hydroxylase, the rate-limiting enzyme in dopamine synthesis, requires iron as a cofactor; a low brain iron state has been repeatedly demonstrated in RLS (26). However, current evidence points to a pre-synaptic hyperdopaminergic state in RLS as revealed by a decreased dopamine transporter activity and a decreased dopamine reuptake leading to a downregulation of postsynaptic D2 dopamine receptors (27). A circadian regulation of dopaminergic activity, i.e., a nadir at night and a peak in the morning, may explain why dopaminergic drugs are effective in the treatment of restless legs symptoms in the presence of a hyperdopaminergic state (28). However, by further downregulating the dopamine receptors, medication at night might paradoxically worsen restless legs symptoms, a phenomenon known as augmentation which is the most important long-term adverse effect of dopaminergic therapy (29).

Although the underlying mechanisms are not fully understood, epidemiological studies suggest that psychiatric disorders and RLS may frequently co-occur, leading to complex interactions between both conditions (25, 30, 31). A bidirectional cause-and-effect relationship is postulated, however, the available evidence is scarce (25). Particularly, comorbid anxiety and depressive disorders are major factors influencing the course of RLS and quality of life of affected patients (25). Previous studies found that treatment outcomes of RLS with comorbid depression are worse compared to RLS without depressive symptoms (32). Furthermore, longitudinal studies have demonstrated an increased risk of depression in RLS patients and, vice versa, an increased likelihood of the occurrence of RLS in depression, suggesting a bidirectional relationship between both disorders (33). Moreover, patients with mental disorders often require treatment with psychotropic drugs that are associated with an increased risk to induce or exacerbate RLS (21–23). However, an increased risk of depression in patients with RLS may also be independent of psychopharmacological effects (34).

Despite the close relationship between RLS and psychiatric disorders, studies on the prevalence of RLS in psychiatric patients are scarce and refer to limited patient samples in Asia (35–37) or otherwise strongly selected psychiatric samples (38, 39). The aim of the present study was to estimate the prevalence and assess the clinical correlates of RLS in a larger sample of psychiatric inpatients in Germany and Switzerland.

## Materials and Methods

### Study Design and Participants

The multicenter cross-sectional study, which was initiated by sleep medicine experts of the German Association for Psychiatry, Psychotherapy, and Psychosomatics, was conducted at five psychiatric hospitals in Germany and two hospitals in Switzerland. The study protocol was approved by the local ethics committees of each participating site and was conducted in accordance with the ethical guidelines of the current version of the Declaration of Helsinki. Recruitment of study participants was consecutive in the period from February 2019 to December 2020. Eligible participants were randomly selected and invited to participate in the study. Patients who were approached and expressed willingness to participate were fully informed about the purpose of the study, and written informed consent was obtained from all participants. Participants of any gender and with an age above 18 years with a psychiatric disorder classified in the International Classification of Diseases (ICD-10) were included in the study (40). Exclusion criteria were severe psychotic disorders or significant cognitive impairment preventing the subject to fully understand the nature of the study, acute suicidality, involuntary admission to the hospital, and lack of capacity to understand the purpose of the study. Known or treated RLS was not an exclusion criterion.

### Assessments

Sociodemographic and biometric data including gender, age, and education, as well as health information, were obtained at the time of enrollment. Psychiatric and comorbid medical disorders including non-organic and organic sleep disorders according to the criteria of the ICD-10 classification were assessed based on medical history and clinical examination and were documented in the case report form. In addition, the use of the following psychiatric medications was recorded: antidepressants [selective serotonin reuptake inhibitors (SSRI), selective serotonin and noradrenaline reuptake inhibitors (SSNRI), tricyclic antidepressants (TCA), mirtazapine, agomelatine, and others], antipsychotics (atypical and typical), hypnotics (benzodiazepines, non-benzodiazepines), mood stabilizers (lithium, lamotrigine, valproate, carbamazepine), anticonvulsants (pregabalin, gabapentin), opioids, and stimulants.

#### RLS Diagnosis

Symptoms of RLS were assessed face-to-face by an RLS expert and were based on the five essential diagnostic criteria defined by the International RLS Study Group (IRLSSG) (1): (1) an urge to move the legs usually but not always accompanied by, or felt to be caused by, uncomfortable and unpleasant sensations in the legs; (2) the urge to move the legs and any accompanying unpleasant sensations beginning or worsening during periods of rest or inactivity such as lying down or sitting; (3) are partially or totally relieved by movement, such as walking or stretching, at least as long as the activity continues; (4) the urge to move the legs and any accompanying unpleasant sensations during rest or inactivity only occur or are worse in the evening or night than during the day; and (5) the presence of the above features cannot solely be accounted for as symptoms primary to another medical or a behavioral condition. According to criterion 5, a diagnosis of RLS is made after careful consideration of differential diagnoses and the exclusion of mimicking conditions such as leg cramps, positional discomfort, anxiety, or drug-induced akathisia.

#### RLS Severity

In the case of an RLS diagnosis, the intensity, duration, and frequency of RLS symptoms, as well as their impact on sleep, daytime well-being, and mood, were assessed using the International Restless Legs Scale (IRLS), a rating scale developed by the IRLSSG. This scale includes 10 items on a five-point Likert scale. RLS severity is classified as mild with a score of 1–10, moderate with 11–20, severe with 21–30, and very severe with 31–40 (41).

#### Sleep Quality

Sleep quality was evaluated by the Pittsburgh Sleep Quality Index (PSQI) (42). The PSQI is a 19-item questionnaire that assesses subjective sleep quality including sleep latency, duration, disturbances, and daytime dysfunction. PSQI global sleep quality scores are continuous; the total PSQI score ranges from 0 to 21 points, with a score above 5 indicating poor sleep quality.

#### Insomnia

Insomnia was measured using the Insomnia Severity Index (ISI), a seven-item questionnaire that assesses difficulty falling asleep, nighttime awakenings, early morning awakenings, impairment of daytime functioning, distress and worry about sleep, and current dissatisfaction with sleep (43, 44). Each item is rated on a Likert scale ranging from 0 to 4. The ISI total score ranges from 0 to 28 points and classifies subjects into “no or minimal insomnia,” “subthreshold insomnia,” “moderate insomnia,” and “severe insomnia” groups (45). A score of 7–14 represents subthreshold insomnia; a score above 14 represents manifest insomnia (46).

#### Daytime Sleepiness

To measure daytime sleepiness, the Epworth Sleepiness Scale (ESS), a self-administered 8-item questionnaire, was used (47). The rating for each item is on an ascending scale from 0 to 3. The ESS asks for the likelihood of dozing off or falling asleep in different everyday situations, in contrast to feeling just tired. A total ESS score greater than 10 (range 0–24) is indicative of increased daytime sleepiness.

#### Mood

We used the Patient Health Questionnaire (PHQ-9), a 9-question assessment of the occurrence of depressive symptoms that uses a four point Likert scale (0: not at all; 3: nearly every day), to screen for the presence and severity of depression (48–50). Scores from 5 to 9 represent mild depression, 10–14 moderate depression, 15–19 moderately severe depression, and 20 or more severe depression.

### Statistical Analysis

The main outcome parameter was the prevalence of an RLS diagnosis. Exploratory analyses were conducted to determine whether there were diagnosis- or treatment-related differences in the frequency of RLS and whether certain aspects of the socio- or biometric data were associated with the occurrence of RLS. Furthermore, differences in daytime sleepiness, insomnia symptoms, and sleep quality between patients with and without RLS were analyzed. For variables assessed at an ordinal or nominal scale, descriptive statistics are provided as frequencies in absolute numbers and percentages. For those variables, Cramer’s V is used as an effect size measure, where values less than 0.3 indicate a small effect, values from 0.3 to 0.4 a medium effect, and greater than 0.4 a large effect. Differences in the distribution of variables between patients with and without RLS were assessed using a Likelihood-ratio chi-square test if the number of characteristic expressions exceeded two; if the number was equal to two, Fisher’s exact test was used. Odds ratios (OR) and their 95% confidence intervals (95% CI) were calculated to quantify the strengths of associations. For continuously measured variables, the mean and its standard deviation as well as the median and the interquartile range are given as descriptive statistics. Cohen’s d was calculated as a measure of effect size, where values less than 0.5 indicate a small effect, values from 0.5 to 0.8 a medium effect, and values greater than 0.8 a large effect. Because all continuously measured variables did not follow a Gaussian distribution as assessed by a Shapiro–Wilk test, differences in distributions between patients with and without RLS were assessed non-parametrically using the Wilcoxon Two-sample test. All tests were performed with a double-sided *p* (<0.05).

To identify significant risk factors for the occurrence of RLS, all variables that showed significant differences in distribution between patients with and without RLS at the univariate level were considered for inclusion in a logistic regression model. After exclusion of highly correlated variables, logistic regression analysis was performed with age, gender, RLS family history, BMI, ESS, and PSQI scores, frequency of alcohol consumption, use of antidepressants, and use of antipsychotics as independent factors. All statistical analyses were performed using SAS software (version 9.4M3; SAS Institute, Cary, NC, United States).

## Results

Overall, 331 patients met the inclusion criteria and were invited to participate in the study. Of these, 14 patients declined the study participation after being fully informed about the purpose of the study. A total of 52 of the 317 patients met the diagnostic criteria of RLS according to the IRLSSG (1), representing a prevalence of 16.4% (95% CI: [12.3; 20.5]). The diagnoses of the patients were classified according to the following ICD-10 codes: F10-19: Mental and behavioral disorders due to psychoactive substance use; F20-29: Schizophrenia, schizotypal, and delusional disorders; F30-39: Mood [affective] disorders; F40-48: Neurotic, stress-related and somatoform disorders; F60-69: Disorders of adult personality and behavior; F90-98: Behavioral and emotional disorders with onset usually occurring in childhood and adolescence. Further sample characteristics of patients with and without RLS for ordinally and nominally scaled variables are given in Table 1. Among patients with RLS, an RLS diagnosis was previously known in 23.1, and 19.2% were treated with an RLS-specific medication. In 54.8% of all patients with RLS, complaints of symptoms had been present for more than 2 years prior to diagnosis and more than 3 years in 28.6% of patients. In women and in first-degree relatives, RLS prevalence was significantly higher in patients with RLS compared to the non-RLS group. 15.7% of the patients with RLS had a positive family history of RLS. In addition, BMI was significantly higher in patients with RLS, while the frequency of alcohol consumption as well as the number of alcoholic drinks per day was significantly lower. Furthermore, PSQI (sleep quality), ISI (insomnia), and ESS (daytime sleepiness) scores were significantly higher in patients with RLS.

**TABLE 1:** Descriptive statistics for variables measured at with ordinal or nominal scale and results of statistical assessment of differences between patients with and without restless legs syndrome (RLS).

	Total (*n* = 317)		No RLS (*n* = 265)		RLS (*n* = 52)					
Variables	*n*	%	*n*	%	*n*	%	p	Cramer’s V	OR	95% CI
*Gender*										
Female	180	56.8	141	53.2	39	75	**0.0036**	0.1629	**2.64**	**[1.35; 5.15]**
Male	137	43.2	124	46.8	13	25				
**BMI (kgm^–2^)**										
<18.5	15	4.7	15	5.7	0	0	**0.0161**	0.1928		
18.5 ≤ BMI < 25.0	142	44.8	118	44.5	24	46.2				
25.0 ≤ BMI < 30.0	85	26.81	77	29.1	8	15.4				
30.0 ≤ BMI < 35.0	48	15.1	35	13.2	13	25				
35.0 ≤ BMI < 40.0	19	6	14	5.3	5	9.6				
40.0 ≤ BMI	8	2.5	6	2.3	2	3.9				
**Education (years)**										
≤10	79	24.9	69	26	10	19.2	0.3811	0.0583	1.47	[0.70; 3.11]
>10	238	75.1	196	74	42	80.8				
**Primary psychiatric diagnosis (ICD-10)**										
F10-19: Mental and behavioral disorders due to psychoactive substance use	58	18.3	54	20.4	4	7.7	0.2043	0.1387		
F20-29: Schizophrenia, schizotypal and delusional disorders	30	9.5	25	9.4	5	9.6				
F30-39: Mood [affective] disorders	173	54.6	139	52.5	34	65.4				
F40-48: Neurotic, stress-related and somatoform disorders	30	9.5	26	9.8	4	7.7				
F60-69: Disorders of adult personality and behavior	24	7.6	19	7.2	5	9.6				
F90-98: Behavioral and emotional disorders with onset usually occurring in childhood and adolescence	2	0.6	2	0.8	0	0				
**Secondary psychiatric diagnoses**										
No	153	48.3	124	46.8	29	55.8	0.2882	0.0665		
Yes	164	51.74	141	53.21	23	44.23				
**No. of secondary psychiatric diagnoses**										
0	153	48.3	124	46.8	29	55.8	0.4878	0.0876		
1	86	27.1	76	28.7	10	19.2				
2	47	14.8	40	15.1	7	13.5				
≥3	31	9.8	25	9.4	6	11.5				
**Somatic diagnoses**										
No	151	47.6	127	47.9	24	46.2	0.8798	0.0131	1.07	[0.59; 1.95]
Yes	166	52.4	138	52.1	28	53.9				
Sleep disorders*										
No	231	72.9	193	72.8	38	73.1	1	0.0021	0.99	[0.51; 1.93]
Yes	86	27.1	72	27.2	14	26.9				
**RLS in first-degree relatives (n = 313)**										
No	292	93.3	249	95	43	84.3	**0.0108**	0.1583	**3.56**	**[1.39; 9.11]**
Yes	21	6.7	13	5	8	15.7				
**Psychopharmacological treatment**										
No	39	12.3	34	12.8	5	9.6	0.6475	0.0362	1.38	[0.51; 3.72]
Yes	278	87.7	231	87.2	47	90.4				
**Tobacco use (n = 316)**										
No	137	43.4	115	43.6	22	42.3	1	0.0094	1.05	[0.58; 1.92]
Yes	179	56.7	149	56.4	30	57.7				
**Frequency of alcohol consumption (n = 315)**										
Never	67	21.7	56	21.3	11	21.2	**0.0016**	0.2356		
1/month	90	28.6	64	24.3	26	50				
2–4/month	60	19.1	52	19.8	8	15.4				
2–3/week	31	9.8	28	10.7	3	5.8				
4–6/week	17	5.4	17	6.5	0	0				
Daily	50	15.9	46	17.5	4	7.8				
No. of alcoholic drinks/day (n = 248)**										
1–2	123	49.6	94	45.2	29	72.5	**0.0081**	0.2356		
3–4	45	18.2	39	18.8	6	15				
5–6	33	13.3	30	14.4	3	7.5				
7–9	10	4	10	4.8	0	0				
≥10	37	14.9	35	6.8	2	5				
**ESS (n = 315)**										
≤10	227	72.1	199	75.7	28	53.9	**0.0022**	0.1805	2.67	[1.44; 4.94]
>10	88	28	64	24.3	24	46.2				
**PSQI (n = 304)**										
≤*5*	43	14.1	41	16.1	2	4	**0.0249**	0.1292	4.62	[1.08; 19.76]
>*5*	261	85.1	213	83.9	48	96				
**ISI (n = 313)**										
≤7	63	20.1	61	23.3	2	3.9	**0.0009**	0.1783	**7.44**	**[1.76; 31.47]**
>7	250	79.9	201	76.7	49	96.1				
≤14	154	49.2	139	53.1	15	29.4	**0.0021**	0.1746	**2.71**	**[1.42; 5.19]**
>14	159	50.8	123	47	36	70.6				
**PHQ-9 (n = 315)**										
≤4	19	6	15	5.7	4	7.8	0.0951	0.1321		
5 ≤ PHQ-9 ≤ 9	49	15.6	46	17.4	3	5.9				
10 ≤ PHQ-9 ≤ 14	65	20.6	56	21.2	9	17.7				
PHQ-9 ≥ 15	182	57.8	147	55.7	35	68.6				

Effect size measure, Cramer’s V; OR, odds ratio; 95% CI, 95% confidence interval of the odds ratio; BMI, body mass index; ESS, Epworth Sleepiness Scale; PSQI, Pittsburgh Sleep Quality Index; ISI, Insomnia Severity Index; PHQ-9, Patient Health Questionnaire; p, Log-Likelihood χ^2^ test p-value or Fisher’s exact p, respectively. *Sleep disorders according to ICD-10: F51.0: Non-organic insomnia; F51.1: Non-organic hypersomnia; F51.2: Non-organic disorder of the sleep-wake schedule; F51.3 Sleepwalking (somnambulism); F51.4Sleep terrors (night terrors); F51.5: Nightmares; G25.80: Periodic leg movements in sleep; G47.0: Disorders of initiating and maintaining sleep (insomnias); G47.1: Disorders of excessive somnolence (hypersomnias); G47.2: Disorders of the sleep-wake schedule; G47.3: Sleep apnea; G47.4: Narcolepsy and cataplexy; G47.8: Other sleep disorders; G25.8: RLS was explicitly not taken into account. **A standard drink contains 10–12 g of alcohol (i.e., 0.33l of regular beer, 0.125l of wine or 4cl shot of distilled spirits). Statistically significant results are shown in bold.

No differences between patients with and without RLS were observed for the distribution of primary and secondary psychiatric diagnoses, somatic and sleep medicine diagnoses, or for current psychopharmacological therapy (except for antipsychotics), smoking, educational status, or PHQ-9-scores (Table 1).

Sample characteristics related to the distribution of continuous variables are reported in Table 2. Although patients with RLS tended to be slightly older than patients without RLS, this difference was not significant. Statistically significant differences in the prevalence of patients with increased daytime sleepiness, impaired sleep quality, and insomnia symptoms are reflected in significant differences between patients with and without RLS in the overall distribution of these scores. No significant differences were observed in age at psychiatric diagnosis, duration of psychiatric illness, smoking habits, or the distribution of PHQ-9 scores.

**TABLE 2:** Descriptive statistics for variables measured at a continuous scale and results of statistical assessment of differences between patients with and without restless legs syndrome (RLS).

Variables	Total (*n* = 317)		No RLS (*n* = 265)		RLS (*n* = 52)		d	*p*
	Mean ± SD	Median [IQR]	Mean ± SD	Median [IQR]	Mean ± SD	Median [IQR]		
Age (years)	42.2 ± 15.3	41 [29; 55]	41.6 ± 15.4	40 [29; 53]	45.8 ± 14.4	49 [34; 57]	0.28	0.0512
Age at psychiatric diagnosis (years) (n_*T*_ = 312, n_*RLS–*_ = 260, n_*RLS+*_ = 52)	33.0 ± 14.4	31 [21; 42]	32.6 ± 14.1	30 [21; 40]	35.0 ± 15.5	33 [22; 46]	0.17	0.3255
Duration of illness (years after diagnosis) (n_*T*_ = 312, n_*RLS–*_ = 260, n_*RLS+*_ = 52)	9.2 ± 10.2	6 [1; 15]	8.9 ± 9.9	5 [1; 15]	10.7 ± 11.7	7 [2; 17]	0.18	0.2881
PSQI (n_*T*_ = 304, n_*RLS–*_ = 254, n_*RLS+*_ = 50)	10.6 ± 4.7	10 [7; 15]	10.2 ± 4.7	10 [6; 14]	12.7 ± 4.3	14 [9; 16]	1.29	**0.0007**
ISI (n_*T*_ = 313, n_*RLS–*_ = 262, n_*RLS+*_ = 51)	14.1 ± 6.9	15 [9; 19]	13.5 ± 7.0	13.5 [8; 19]	17.2 ± 5.3	18 [13; 15]	0.56	**0.0003**
ESS (n_*T*_ = 315, n_*RLS–*_ = 263, n_*RLS+*_ = 52)	7.7 ± 4.8	7 [4; 11]	7.3 ± 4.8	7 [3; 10]	9.7 ± 4.2	10 [6; 13]	0.51	**0.0005**
PHQ-9 (n_*T*_ = 315, n_*RLS–*_ = 264, n_*RLS+*_ = 51)	15.2 ± 6.6	16 [10; 20]	15.0 ± 6.4	16 [10; 20]	16.4 ± 6.1	17 [13; 20]	0.22	0.1684
No. of cigarettes/day (n_*T*_ = 178, n_*RLS–*_ = 148, n_*RLS+*_ = 30)	17.3 ± 10.6	15 [10; 15]	17.6 ± 10.7	17.5 [10.0; 20.0]	15.7 ± 10.4	12.5 [10.0; 20.0]	0.18	0.3057
No. of smoker years (n_*T*_ = 205, n_*RLS–*_ 170, n_*RLS+*_ = 35)	19.7 ± 13.1	30 [18; 40]	19.5 ± 12.0	19 [8; 30]	20.8 ± 14.4	17 [8; 30]	0.10	0.7296

*SD, standard deviation; IQR, interquartile range; d, effect size Cohen’s d (small effect: d < 0.5; medium effect: 0.5 ≤ d < 0.8; large effect: ≥ 0.8), n_T_, number of total; n_RLS–_, number of RLS negative patients; n_RLS+_, number of RLS positive patients; PSQI, Pittsburgh Sleep Quality Index; ISI, Insomnia Severity Index; ESS, Epworth Sleepiness Scale; PHQ-9, Patient Health Questionnaire; since all parameters proofed to be not normally distributed by Shapiro–Wilks test, p-value refers to the Wilcoxon-Two-Sample test. Statistically significant results are shown in bold.*

**FIGURE 1:**
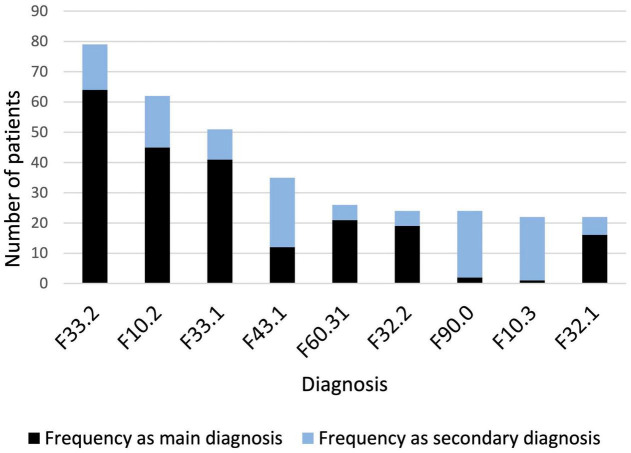
Diagnoses with a cumulative frequency > 20. F33.2: Recurrent depressive disorder, current episode severe without psychotic symptoms; F10.2: Mental and behavioral disorders due to use of alcohol, dependence syndrome; F33.1: Recurrent depressive disorder, current episode moderate without psychotic symptoms; F43.1: Posttraumatic stress disorder; F60.31: Emotionally unstable personality disorder, borderline; F32.2: Severe depressive episode; F90.0: Attention deficit/hyperactivity disorder; F10.3: Mental and behavioral disorders due to use of alcohol, withdrawal state; F32.1: Moderate depressive episode.

Overall, there was a wide diversity of primary and secondary psychiatric diagnoses. To assess the association between specific psychiatric diagnoses and RLS, only diagnoses with a cumulative frequency of more than 20 were considered; this resulted in 9 concomitant diagnoses in the data set. The frequencies of the corresponding diagnoses according to ICD-10 are shown in Figure 1.

Treatment with antidepressants, hypnotics, mood stabilizers, or anticonvulsants (when considered at the class level and at the level of specific drugs), as well as with opiates or stimulants were not associated with RLS (Supplementary Table 2). A statistically significant association between pharmacotherapy and RLS was observed only for treatment with antipsychotics (*p* = 0.0491). RLS patients received antipsychotics more often than patients without RLS. When atypical and typical antipsychotics were analyzed separately, the effect was restricted to atypical antipsychotics (*p* = 0.0317; Supplementary Table 2).

Restless legs syndrome severity according to the IRLS was reported between moderate and severe (mean 20.3; median 22). A comorbidity or secondary RLS was affirmed 21 times, allowing multiple answers. Thyroid disorders were the most frequent comorbidity (15.4%, *n* = 8) followed by drug-induced RLS (13.5%, *n* = 7). In addition, drug-induced worsening of RLS was reported in 17.7% (n = 9) of cases. Further details are reported in Tables 3, 4.

**TABLE 3:** Restless legs syndrome (RLS) characteristics for variables measured on a continuous scale.

Variables	Mean ± SD	Median [IQR]	95% CI
Duration (months) of symptoms in newly diagnosed RLS (*n* = 29)	70.1 ± 134.8	30 [12;72]	[18.9;121.4]
Duration (months) of symptoms before diagnosis with known RLS (*n* = 12)	62.7 ± 87.2	42 [7;78]	[7.3;118.1]
Number of definable episodes (*n* = 10)	13.1 ± 30.7	3 [2;6]	[-8.8;35.0]
IRLS (*n* = 47)	20.3 ± 8.4	22 [14;26]	[17.8;22.8]

*IRLS, International RLS Severity Scale.*

**TABLE 4:** Restless legs syndrome (RLS) characteristics for variables measured at an ordinal or nominal scale.

Variables	Frequency (%)
**RLS diagnosis known before study begin**	
No	76.9
Yes	23.1
**Duration of RLS (n = 42)**	
≤6 months	23.8
6 < duration ≤ 12 months	11.9
12 < duration ≤ 24 months	9.5
24 < duration ≤ 60 months	26.2
>60 months	28.6
**Newly diagnosed RLS, duration (n** = *30)*	
≤6 months	23.3
6 < duration ≤ 12 months	10.0
12 < duration ≤ 24 months	13.3
24 < duration ≤ 60 months	23.3
>60 months	30.0
**Previously diagnosed RLS, duration (n** = *12)*	
≤6 months	25.0
6 < duration ≤ 12 months	16.7
12 < duration ≤ 24 months	0.0
24 < duration ≤ 60 months	33.3
>60 months	25.0
**Frequency of RLS (n** = *52)*	
<1×/month	7.7
1–3×/month	7.7
1–2×/week	25.0
3–6×/week	30.8
Daily	28.9
**Iron deficiency**	
No	75.0
Yes	5.8
Unknown	19.2
**Kidney disease**	
No	96.2
Yes	1.9
Unknown	1.9
**Polyneuropathy**	
No	96.2
Yes	1.9
Unknown	1.9
**Thyroid disorder**	
No	82.7
Yes	15.4
Unknown	1.9
**Pregnancy**	
No	89.1
Yes	1.9
Unknown	0.0
**Drug-induced RLS**	
No	78.9
Yes	13.5
Unknown	7.7
**Drug-induced worsening of RLS (N = *51)***	
No	68.6
Yes	17.7
Unknown	13.7
**Drug treatment of RLS**	
No	80.8
Yes	19.2
Unknown	0.0

**FIGURE 2:**
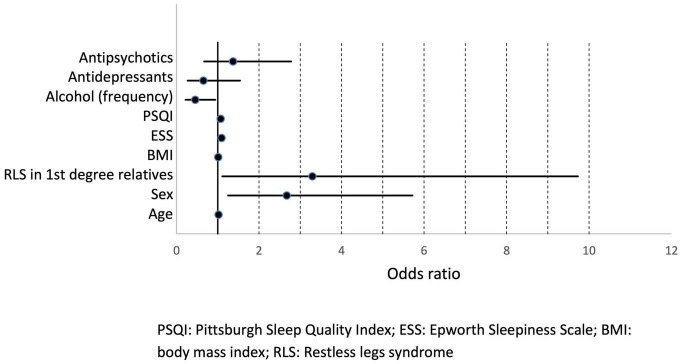
Odds ratios with 95% confidence intervals. PSQI, Pittsburgh Sleep Quality Index; ESS, Epworth Sleepiness Scale; BMI, body mass index; RLS, restless legs syndrome.

**FIGURE 3:**
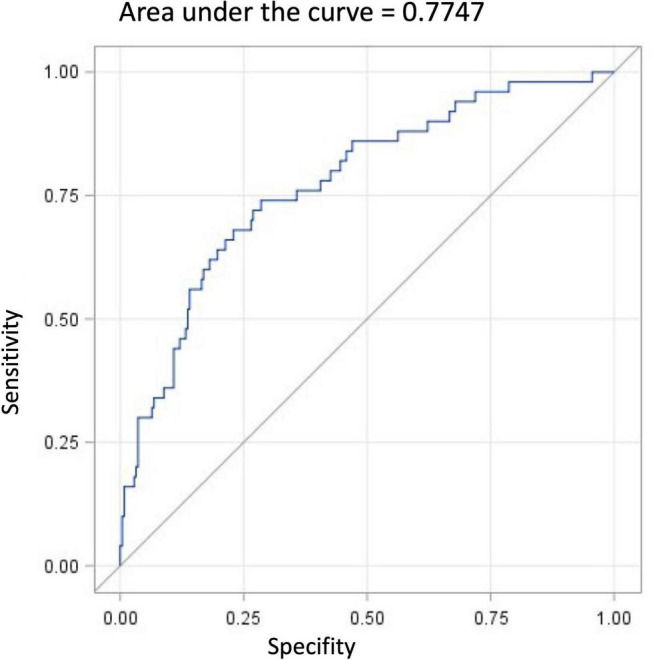
Receiver operating characteristic (ROC) curve of logistic regression model.

To identify significant predictors for the occurrence of RLS, all variables that showed significant differences in distribution between patients with and without RLS at the univariate level were considered for inclusion in a logistic regression model. After exclusion of highly correlated variables, logistic regression analysis was performed and included age, gender, RLS family history, BMI, ESS, and PSQI scores, frequency of alcohol consumption, use of antidepressants, and use of antipsychotics as independent factors (Table 5). Of these, age, gender, positive family history, and ESS score were statistically associated with higher risk of RLS. Psychiatric patients with first-degree relatives who have RLS had the highest RLS risk (OR 3.29, 95% CI [1.1; 9.7]). Women had a 2.7-fold higher risk (95% CI: [1.25; 5.72]) compared to men. Increased daytime sleepiness was also associated with the occurrence of RLS (OR 1.09, 95% CI: [1.01; 1.17]). On the other hand, a higher frequency of alcohol consumption was associated with a lower risk of RLS (OR 0.45, 95% CI: [0.22; 0.94]). Age, BMI, PSQI score, and the use of antidepressant or antipsychotic medications were not significantly associated with an increased RLS risk (Figure 2). A receiver operating characteristic (ROC) curve resulted in an area under the curve of 0.7747, which corresponds to an acceptable discrimination between the RLS yes/no conditions (Figure 3).

**TABLE 5:** Results of logistic regression analysis of 299 patients for occurrence of restless legs syndrome (RLS; 50 patients with and 249 without RLS).

Variable/predictor	β	SE β	Wald’s χ^2^	df	*p*	OR	OR [95% CI]
Constant	−3.4720	1.1537	9.0571	1	0.0026	NA	
Age (years)	0.0202	0.0116	3.0072	1	0.0829	1.02	[1.00; 1.04]
Gender (default = male, 1 = female)	0.4909	0.1942	6.3867	1	**0.0115**	**2.67**	**[1.25; 5.72]**
RLS in first-degree relatives (default = no; 1 = yes)	0.5952	0.2769	4.6207	1	**0.0316**	**3.29**	**[1.11; 9.73]**
BMI (kgm^–2^)	0.0103	0.0261	0.1563	1	0.6926	1.01	[0.96; 1.06]
ESS	0.0814	0.0372	4.8028	1	**0.0284**	**1.09**	**[1.01; 1.17]**
PSQI	0.0700	0.0393	3.1658	1	0.0752	1.07	[0.99; 1.16]
Frequency of alcohol consumption (2 = ≤ 1/month; 1 = > 1/month)	−0.3981	0.1880	4.4828	1	**0.0342**	**0.45**	**[0.22; 0.94]**
Antidepressants (2 = no; 1 = yes)	−0.4294	0.4423	0.9424	1	0.3317	0.65	[0.27; 1.54]
Antipsychotics (2 = no; 1 = yes)	0.1557	0.1809	0.7410	1	0.3893	1.37	[0.67; 2.78]

**Test**			**χ^2^**	**df**	***p***		

Overall model evaluation Likelihood ratio test			44.4938	9	<0.0001		
Score test			42.6423	9	<0.0001		
Wald test			33.8414	9	<0.0001		

*BMI, body mass index; ESS, Epworth Sleepiness Scale; PSQI, Pittsburgh Sleep Quality Index; ISI, Insomnia Severity Index; β, regression coefficient, SE β, standard error of the regression coefficient; df, degrees of freedom, p, p-value of Wald’s χ^2^ OR, odds ratio; 95% CI, 95% confidence interval of the odds ratio. Statistically significant results are shown in bold.*

## Discussion

In the present multicenter study, an RLS prevalence of 16.4% was observed in a sample of 317 psychiatric inpatients, which is markedly higher than the RLS frequency in the general population, which is estimated to be 5–10% (2, 12, 51). According to the IRLS, RLS in our patients was moderate to severe. A prior study in a smaller sample of 117 hospitalized psychiatric patients reported an RLS prevalence of 19.7% (39). In contrast to the present study, only patients with comorbid severe sleep disturbances such as insomnia, dyssomnia or daytime sleepiness were included in this retrospective analysis, precluding direct comparison with the present results. In a Spanish cross-sectional study of a sample of 100 non-demented psychogeriatric outpatients (mean age 76.9 ± 6 years), a prevalence of 11.1% was shown for definite RLS with an additional prevalence of 10.1% for possible RLS. Because of the specific age range of the included patients (38), there is no direct comparability with the present study sample (mean age 42.2 ± 15.3), as RLS prevalence is age-dependent (2). Studies in Lebanon revealed RLS prevalence rates of 14.3 (37) and 18.0% (35) in a sample of 203 and 126 hospitalized psychiatric patients, respectively. Both studies used the IRLS not only as a tool to determine the severity of RLS, but also as a diagnostic screening instrument. However, the scale is only validated as an instrument for determining severity and not for diagnosing RLS (41), clearly limiting the results of these studies. A more recent study in Singapore yielded a prevalence of RLS/PLMS (periodic leg movements during sleep) of 14.8% in a sample of 400 psychiatric outpatients with schizophrenia, mood disorders, or anxiety disorders, based on ICD-9 diagnostic criteria (36). In this study, two out of four positive responses on RLS-related questions were considered sufficient for an RLS diagnosis. In addition, the criteria according to the IRLSSG were not applied. Comparability of these results with the present study is highly limited due to methodological differences and limitations. In addition, RLS prevalences have been shown to be much lower in Asia (0.9–7.2%; overview in Ref. (3) than that determined by Hombali et al. (36).

Among the psychiatric inpatients with RLS included the present study, a high proportion (76.9%) were previously undiagnosed, although for 54.8% of the patients, symptoms had been present for more than 2 years. It has been repeatedly demonstrated that RLS is often underdiagnosed and untreated (52, 53) because symptoms are primarily subjective and are based almost exclusively on patient statements. Affected persons may have difficulty adequately describing the unpleasant sensations as well as the urge to move. Often, patients with RLS do not complain of restless legs symptoms, but of insomnia or fatigue, leading the symptoms of RLS to be falsely attributed to a mental condition (54, 55). Specifically in a psychiatric context, the differentiation of RLS complaints and neuroleptic-induced akathisia may be challenging (56).

Patients with a first-degree relative with RLS symptoms were significantly more likely to have RLS themselves. Familial clustering of RLS is a frequent finding (9), which can be interpreted as an expression of increased genetic vulnerability. Currently, 22 gene loci are known to be associated with an increased incidence of RLS (57, 58). In line with previous epidemiological studies in the general population, we found an increased RLS prevalence for women (21.7 vs. 9.5% in men) (9, 18).

### Smoking and Alcohol Consumption

Lifestyle factors such as smoking and higher alcohol consumption may be associated with an increased risk for RLS (19, 34, 58, 59). In the present sample, we found no association between smoking and RLS. Nicotine has dopamine-stimulating effects that potentially reduce RLS symptoms (60). However, the association between increased nicotine use and an improvement of RLS has only been published in case reports (61, 62). Another case–control study reported an association between nocturnal smoking and the occurrence of RLS symptoms in 12.0% of patients with RLS and 2.0% in control participants; patients with RLS were more likely to have a comorbid mental disorder. Smoking behavior was also discussed by the authors as an expression of counter regulation with respect to RLS (63). It is well established that psychiatric patients have higher nicotine use compared with the general population (64, 65). In line with these findings, the present sample of patients with RLS reported a higher percentage of smoking (56.5%) compared to the general population (65). Overall, the findings suggest a complex interaction between RLS, smoking, and psychiatric disorders, which has not been studied systematically.

It has been reported that alcohol consumption may induce or exacerbate RLS symptoms (58, 66, 67), while other studies have not found such an association (68–70) or have found an even lower risk of RLS (71, 72), suggesting a potential protective effect. In the present study, patients with RLS reported moderate alcohol consumption in terms of both frequency and intensity. Interestingly, a polymorphism in the alcohol dehydrogenase 1B (*ADH1B*) gene has been identified, which is associated with an increased risk for RLS and low alcohol consumption (73). The relationship between RLS and alcohol is also complicated by the fact that RLS can be triggered by alcohol withdrawal (74). In the present study sample that includes patients with alcohol dependency (ICD-10 Code F10.2) as well as alcohol withdrawal syndrome (ICD-10 Code F10.3), a trend was found for a lower prevalence of RLS among patients with an alcohol use disorder compared to those with another psychiatric disorder. Due to methodological limitations and the cross-sectional design of the present study, causal conclusions cannot be drawn. However, it is of major clinical and scientific interest to clarify the relationship between alcohol consumption and the manifestation of RLS.

### Psychotropic Drugs

Treatment with psychotropic drugs including antidepressants, hypnotics, mood stabilizers and anticonvulsants, opiates, and stimulants was not associated with RLS. No correlation between the number of psychotropic drugs and the occurrence of RLS could be demonstrated. Significant distribution differences were found for the category “antipsychotics” and the subcategory “atypical antipsychotics,” which were taken more frequently by patients with RLS than those without. In agreement with the results of the present study, the use of antipsychotics is associated with an increased risk of RLS (23, 75, 76), which may be due to a drug-induced interference with dopamine metabolism. However, the concept of altered dopamine function as the basis of RLS seem to be incomplete (77). In addition to several genetic variants involved with neural development and brain metabolism, there is evidence for a state of spinal and supraspinal hyperexcitability (78), and recent data have demonstrated alterations of glutamatergic and adenosine signaling in the pathophysiology of RLS (27). Therefore, pharmacological effects interfering with a highly complex system should be interpreted cautiously. In our study, the selection of psychotropic drugs was based on clinical symptoms and not under controlled conditions. An increased awareness at the study sites for the induction of RLS symptoms may also play a role. Dosages of the medications were not assessed, although a dose-dependent effect can often be observed in clinical practice. Randomized controlled trials investigating the association of psycho-pharmacotherapy and RLS are lacking, and most publications are based on single case studies or small case series (22, 23). In a prospective study, RLS occurred or worsened in 9% of patients during treatment with second-generation antidepressants with mirtazapine presenting the highest risk of RLS (28% of cases) (79). Current categorizations of the RLS risk with specific psychotropic drugs are based on expert opinion [overview in Ref. (25)]. A few psychotropic drugs may have a favorable effect on RLS such as aripiprazole (80, 81), bupropion (82), or benzodiazepines (83). However, due to limited evidence, no recommendation can be made for routine clinical use of psychotropic drugs in the treatment of RLS (84).

Non-psychotropic drugs that were not recorded in our study may also induce or exacerbate RLS (85). A drug-independent association of RLS with mental conditions has also been discussed (2, 30, 31). Accordingly, a recent study of Danish blood donors demonstrated that RLS prevalence was increased in people with depression untreated by medication (34).

### Psychiatric Diagnosis and RLS

Approximately every second patient (51.7%) had at least one psychiatric diagnosis in addition to the primary diagnosis, and approximately every fourth patient (24.6%) had three or more psychiatric diagnoses. We found no differences between patients with and without RLS regarding the distribution of primary and secondary psychiatric diagnoses. Increased odds ratios were observed for the association between RLS and posttraumatic stress disorder (PTSD), but not for other psychiatric disorders such as depression. Accordingly, it is very challenging to map associations of RLS with a specific diagnosis. With regard to somatic or sleep medical diagnoses apart from RLS, no differences were found between patients with and without RLS. However, it must be taken into account that these diagnoses were not systematically explored for the purpose of the present study, and diagnoses were taken from the patients’ records.

### Sleep Quality and Mood

Subjective sleep quality was markedly impaired in patients with RLS as reflected by a significantly higher mean PSQI score (12.7) compared to the non-RLS group (10.2). Disturbed sleep is a characteristic feature in RLS; however, because no polysomnographic data were available in the present study, it cannot be differentiated whether the sleep problems are due to restless legs symptoms, concomitant periodic leg movements, or possibly due to another comorbid sleep disorder. Insomnia symptoms as measured by the ISI were significantly higher in RLS patients. Approximately 50% of all patients with RLS reported an ISI score above 14, but only about 20% showed an ISI score less than 7, demonstrating that the vast majority of psychiatric patients have complaints of at least mild insomnia. The manifestation of RLS in the form of insomnia symptoms is of great clinical importance, because sleep disturbances are a core symptom of various mental disorders. Therefore, a detailed sleep-related exploration including RLS criteria is essential.

Epworth Sleepiness Scale scores, reflecting daytime sleepiness, were also higher in the RLS patient group; however, this group’s mean score of 9.7 is below the pathological cut-off score. According to the IRLSSG, a lack of pronounced daytime sleepiness is regarded as a supportive RLS diagnostic criterion (1). However, 50% of the patients with RLS showed ESS scores above 10, which is indicative of increased daytime sleepiness. It remains unclear whether these patients had an additional specific sleep disorder, such as sleep apnea, so substantial conclusions cannot be drawn. A previous multicenter study on a comparable clinical sample found a high prevalence (21.7%) of previously unknown obstructive sleep apnea syndrome (OSAS) in 298 psychiatric inpatients (86). An increased comorbidity of RLS in patients with OSAS has also been demonstrated, especially in patients with insomnia symptoms (87, 88).

### Limitations

The present study has several limitations that should be considered. First, the diagnosis of RLS was based on the five minimal IRLSSG criteria (1); although careful clinical examinations were performed to exclude RLS mimics, no additional technical diagnostic tests were conducted. Therefore, it is possible that the reported prevalence is an overestimation. However, the vast majority of RLS prevalence studies in the general population are based on the former four or five essential RLS criteria without additional diagnostic tests (2, 3). Compared with these prevalence rates, the prevalence of RLS in our sample is most likely slightly inflated. The challenge in correctly diagnosing RLS lies in improving the specificity of the symptoms (55, 89), which is currently viewed as critical (90). Second, sleep quality was not evaluated by objective sleep measurements such as actigraphy or polysomnography, therefore sleep disturbing PLMS associated with RLS or sleep-related breathing disorders could not be detected. Third, somatic and sleep medicine diagnoses were taken from the patients’ records. Fourth, the sample size was probably too small to detect drug-related effects on RLS beyond the association with atypical neuroleptics. However, no consistent classification of drugs in terms of favorable or adverse effects on restless legs symptoms is feasible that would be a prerequisite for definite conclusions in cases of polypharmacy.

## Conclusion

In this prospective, cross-sectional, multicenter study, RLS was common among psychiatric inpatients. RLS was associated with higher BMI, impaired subjective sleep quality, and lower alcohol consumption. In a logistic regression analysis, after adjusting for confounders, gender, a positive family history of RLS, and increased daytime sleepiness were identified as predictors for the occurrence of RLS, whereas a higher frequency of alcohol consumption was associated with a lower frequency of occurrence. Knowledge of the prevalence of RLS appears important to increase awareness and systematic diagnostic assessment, potentially contributing to improved care for psychiatric patients.

## Data Availability Statement

The raw data supporting the conclusions of this article will be made available by the authors, without undue reservation.

## Ethics Statement

The studies involving human participants were reviewed and approved by the Ethics Committees of the Universities of Regensburg, Berlin, Leipzig, Freiburg, Nuremberg, Heidelberg (Germany), and Bern (Switzerland). The patients provided their written informed consent to participate in this study. The patients/participants provided their written informed consent to participate in this study.

## Author Contributions

FW, HD-H, LF, NM, KR, CS, CN, and TW contributed to conception and design of the study. FW, HD-H, ED-S, LF, AH, NM, CM, DN, KR, CS, MSe, MSp, CN, and TW acquired data, performed investigations, and wrote and reviewed the manuscript. HD-H and FW performed quality control, data entry, and statistical analysis. FW, HD-H, and TW wrote the original manuscript draft. All authors have read and approved the final manuscript.

## Conflict of Interest

The authors declare that the research was conducted in the absence of any commercial or financial relationships that could be construed as a potential conflict of interest.

## Publisher’s Note

All claims expressed in this article are solely those of the authors and do not necessarily represent those of their affiliated organizations, or those of the publisher, the editors and the reviewers. Any product that may be evaluated in this article, or claim that may be made by its manufacturer, is not guaranteed or endorsed by the publisher.
