# Supplementary material for: Restless Legs Syndrome Prevalence and Clinical Correlates Among Psychiatric Inpatients: A Multicenter Study
Source: Front Psychiatry. 2022 Mar 14;13:846165. doi: 10.3389/fpsyt.2022.846165 (PMC8967168; doi:10.3389/fpsyt.2022.846165)
Supplement: Supplementary file 1 [file Table_1.docx]

**Supplementary Table S1:** Descriptive statistics for the most common diagnoses (with ICD-10 Codes) and results of statistical assessment of differences between patients with and without RLS

|  | Total  (n = 317) | | No RLS  (n = 265) | | RLS  (n = 52) | |  |  |  |  |
| --- | --- | --- | --- | --- | --- | --- | --- | --- | --- | --- |
| Variables | n | % | n | % | n | % | p | Cramer’s V | OR | 95% CI |
| Alcohol dependency (F10.2)  *No*  *Yes* | 255  62 | 80.4  19.6 | 210  55 | 79.2  20.8 | 45  7 | 86.5  13.5 | 0.2569 | 0.0681 | 0.59 | [0.25; 1.39] |
| Alcohol withdrawal (F10.3)  *No*  *Yes* | 295  22 | 93.1  6.9 | 245  20 | 92.5  7.5 | 50  2 | 96.2  3.9 | 0.5496 | 0.0539 | 0.49 | [0.11; 2.16] |
| Moderate depressive episode (F32.1)  *No*  *Yes* | 295  22 | 93.1  6.9 | 248  17 | 93.6  6.4 | 47  5 | 90.4  9.6 | 0.3784 | 0.0466 | 1.55 | [0.55; 4.41] |
| Severe depressive episode (F32.2)  *No*  *Yes* | 293  24 | 92.4  7.6 | 244  21 | 92.1  7.9 | 49  3 | 94.2  5.8 | 0.7776 | 0.0302 | 0.71 | [0.20; 2.48] |
| Recurrent depressive disorder, current episode moderate without psychotic symptoms (F33.1)  *No*  *Yes* | 276  41 | 87.1  12.9 | 231  34 | 87.2  12.8 | 45  7 | 86.5  13.5 | 0.8249 | 0.0070 | 1.06 | [0.44; 2.53] |
| Recurrent depressive disorder, current episode severe without psychotic symptoms (F33.2)  *No*  *Yes* | 238  97 | 75.1  30.6 | 204  61 | 77.0  23.0 | 34  18 | 65.4  34.6 | 0.0821 | 0.0993 | 1.77 | [0.93; 3.35] |
| Posttraumatic stress disorder (F43.2)  *No*  *Yes* | 282  35 | 89.0  11.0 | 240  25 | 90.6  9.43 | 42  10 | 80.8  19.23 | 0.0515 | 0.1158 | **2.29** | **[1.02; 5.10]** |
| Emotionally unstable personality disorder, borderline (F60.3)  *No*  *Yes* | 291  26 | 91.8  8.2 | 243  22 | 91.7  8.3 | 48  4 | 92.3  7.7 | 1.0000 | 0.0082 | 0.92 | [0.30; 2.79] |
| Attention deficit/hyperactivity disorder (F90.0)  *No*  *Yes* | 293  24 | 92.4  7.6 | 243  22 | 91.7  8.3 | 50  2 | 96.2  3.9 | 0.3922 | 0.0624 | 0.44 | [0.10; 1.94] |

effect size measure: Cramer’s V; OR: odds ratio; 95% CI: 95% confidence interval of the odds ratio; p: Fisher’s exact test
