# Supplementary material for: Restless Legs Syndrome Prevalence and Clinical Correlates Among Psychiatric Inpatients: A Multicenter Study
Source: Front Psychiatry. 2022 Mar 14;13:846165. doi: 10.3389/fpsyt.2022.846165 (PMC8967168; doi:10.3389/fpsyt.2022.846165)
Supplement: Supplementary file 2 [file Table_2.DOCX]

**Supplementary Table S2:** Descriptive pharmacotherapy statistics and results of statistical assessment of differences between patients with and without restless legs syndrome (RLS)

|  | Total  (n = 317) | | No RLS  (n = 265) | | RLS  (n = 52) | |  |  |  |  |
| --- | --- | --- | --- | --- | --- | --- | --- | --- | --- | --- |
| Variables | n | % | n | % | n | % | p | Cramer’s V | OR | 95% CI |
| **Antipsychotics**  *No*  *Yes* | 174  143 | 54.9  45.1 | 152  113 | 57.4  42.6 | 22  30 | 42.3  57.7 | **0.0491** | 0.1120 | **1.83** | **[1.01; 3.35]** |
| Atypical antipsychotics  *No*  *Yes* | 184  133 | 58.0  60.8 | 161  104 | 60.8  39.2 | 23  29 | 44.2  55.8 | **0.0317** | 0.1240 | **1.95** | **[1.07; 3.56]** |
| Typical antipsychotics  *No*  *Yes* | 297  20 | 93.7  6.3 | 250  15 | 94.3  5.7 | 47  +5 | 90.4  9.6 | 0.3439 | 0.0602 | 1.77 | [0.61; 5.11] |
| **Hypnotics**  *No*  *Yes* | 238  79 | 75.1  24.9 | 199  66 | 75.1  24.9 | 39  13 | 75.0  25.0 | 1.0000 | -0.0008 | 1.01 | [0.51; 2.00] |
| Benzodiazepines  *No*  *Yes* | 254  63 | 80.1  19.9 | 214  51 | 80.8  19.2 | 40  12 | 76.9  23.1 | 0.5689 | 0.0356 | 1.26 | [0.62; 2.57] |
| Non-Benzodiazepines  *No*  *Yes* | 302  15 | 95.3  4.7 | 251  14 | 94.7  5.3 | 51  1 | 98.1  1.9 | 0.4801 | -0.0586 | 0.35 | [0.05; 2.73] |
| Other hypnotics  *No*  *Yes* | 312  5 | 98.4  1.6 | 260  5 | 98.1  1.9 | 52  0 | 100.0  0.0 | 1.0000 | -0.0561 |  |  |
| **Mood stabilizers**  *No*  *Yes* | 267  50 | 84.2  15.8 | 224  41 | 84.5  15.5 | 43  9 | 82.7  17.3 | 0.6830 | 0.0187 | 1.14 | [0.52; 2.52] |
| Lithium  *No*  *Yes* | 283  34 | 89.3  10.7 | 236  29 | 89.1  11.0 | 47  5 | 90.4  9.6 | 1.0000 | -0.0159 | 0.57 | [0.32; 2.35] |
| Lamotrigine  *No*  *Yes* | 306  11 | 96.5  3.5 | 256  9 | 96.6  3.4 | 50  2 | 96.2  3.8 | 0.6982 | 0.0091 | 1.14 | [0.24; 5.42] |
| Valproate acid  *No*  *Yes* | 307  10 | 96.9  3.1 | 257  8 | 97.0  3.0 | 50  2 | 96.2  3.8 | 0.6710 | 0.0175 | 1.29 | [0.27; 6.23] |
| Carbamazepine  *No*  *Yes* | 317  0 | 100.0  0.0 | 317  0 | 100.0  0.0 | 52  0 | 100.0  0.0 |  |  |  |  |
| **Anticonvulsants**  *No*  *Yes* | 296  21 | 93.4  6.6 | 215  15 | 94.3  5.7 | 46  6 | 88.5  11.5 | 0.0875 | 0.0890 | 2.17 | [0.80; 5.90] |
| Topiramate  *No*  *Yes* | 316  1 | 99.7  0.3 | 265  0 | 100.0  0.0 | 51  1 | 98.1  1.91 | 0.1640 | 0.1270 |  |  |
| Pregabalin  *No*  *Yes* | 300  17 | 94.6  5.4 | 253  12 | 95.5  4.5 | 47  5 | 90.4  9.6 | 0.1704 | 0.0836 | 2.24 | [0.76; 6.66] |
| Gabapentin  *No*  *Yes* | 316  1 | 99.7  0.32 | 264  1 | 99.6  0.4 | 52  0 | 100.0  0.0 | 1.0000 | -0.0249 |  |  |
| **Opiates**  *No*  *Yes* | 304  12 | 96.2  3.8 | 255  10 | 96.3  3.7 | 49  2 | 96.1  3.9 | 1.0000 | 0.0028 | 1.04 | [0.22; 4.90] |
| **Stimulants**  *No*  *Yes* | 307  9 | 97.2  2.8 | 257  8 | 97.0  3.0 | 50  1 | 98.0  2.0 | 1.0000 | -0.0234 | 0.64 | [0.08; 5.25] |

effect size measure: Cramer’s V, V = 0.1: small effect; V = 0.3: medium effect; V = 0.5: large effect; OR: odds ratio; 95% CI: 95% confidence interval of the odds ratio; p: Fisher’s exact test
